# Cardiovascular-kidney-metabolic syndrome in Indonesia: a commentary on the need for integrated management and policy action

**DOI:** 10.3389/fpubh.2025.1707551

**Published:** 2026-01-13

**Authors:** Sally Aman Nasution, Eka Ginanjar, Aida Lydia, Lugyanti Sukrisman, Idrus Alwi, Ika Prasetya Wijaya, Charles Limantoro, Haerani Rasyid, Pringgodigdo Nugroho, Wachid Putranto, Ketut Suastika, Soebagijo Adi Soelistijo, Tri Juli Edi Tarigan, Icha Farihah Deniyati Faratisha

**Affiliations:** 1 Indonesian Society of Internal Medicine, Central Jakarta, Indonesia; 2 Division of Cardiology, Department of Internal Medicine, Faculty of Medicine, Universitas Indonesia, Dr. Cipto Mangunkusumo Hospital, Central Jakarta, Indonesia; 3 Division of Nephrology, Department of Internal Medicine, Faculty of Medicine, Universitas Indonesia, Dr. Cipto Mangunkusumo Hospital, Central Jakarta, Indonesia; 4 Division of Hematology-Oncology, Department of Internal Medicine, Faculty of Medicine, Universitas Indonesia, Dr. Cipto Mangunkusumo Hospital, Central Jakarta, Indonesia; 5 Division of Cardiology, Department of Internal Medicine, Faculty of Medicine, Universitas Diponegoro, Dr. Kariadi Hospital, Semarang, Central Java, Indonesia; 6 Division of Nephrology, Department of Internal Medicine, Faculty of Medicine, Universitas Hasanuddin, Hasanuddin University Hospital, Makassar, South Sulawesi, Indonesia; 7 Division of Nephrology, Department of Internal Medicine, Faculty of Medicine, Universitas Sebelas Maret, The Sebelas Maret University Hospital, Surakarta, Central Java, Indonesia; 8 Division of Endocrinology and Metabolism, Department of Internal Medicine, Faculty of Medicine, Universitas Udayana, Dr. I.G.N.G Ngoerah Hospital, Badung, Bali, Indonesia; 9 Division of Endocrinology and Metabolism, Department of Internal Medicine, Faculty of Medicine, Universitas Airlangga, Dr. Soetomo Hospital, Surabaya, East Java, Indonesia; 10 Division of Endocrinology and Metabolism, Department of Internal Medicine, Faculty of Medicine, Universitas Indonesia, Dr. Cipto Mangunkusumo Hospital, Central Jakarta, Indonesia; 11 Division of Professional Development and Research, Indonesian Society of Internal Medicine, Central Jakarta, Indonesia

**Keywords:** cardiovascular disease, chronic kidney disease, CKM syndrome, Indonesia, metabolic risk, public health, policy

## Abstract

Cardiovascular-kidney-metabolic (CKM) syndrome is a term introduced to reflect the multidirectional relationship between metabolic disorders, kidney disease, and cardiovascular disease (CVD). The prevalence of CKM syndrome, accompanied by its increasing morbidity and mortality, has led researchers, clinicians, and policymakers to undertake synergistic and collaborative efforts in various countries. CKM Syndrome can be categorized into four stages: stage 0 (no risk factors), stage 1 (excessive or dysfunctional adiposity), stage 2 (metabolic risk factors or moderate to high-risk chronic kidney disease/CKD), stage 3 (subclinical cardiovascular disease or CKM syndrome risk equivalents), and stage 4 (clinical CVD in CKM). As the late stage in CKM syndrome, CVD become the leading cause of mortality in Indonesia, accounting for 37–38% of all deaths, with stroke and ischemic heart disease being the predominant causes. Over time, Indonesia has also seen an increase in the prevalence of other illnesses that contribute to the development of CKM syndrome, such as obesity, diabetes mellitus, CKD, hypertension, and dyslipidemia. Rapid urbanization, lifestyle changes, and demographic transitions have amplified the risk of CKM-related conditions. This perspective highlights the emerging risk factors for CKM syndrome in Indonesia, including diabetes mellitus and metabolic syndrome, CKD, and hypertension. This study relies on secondary data from national surveys, government policy reports, and scientific studies related to CKM syndrome. Data were selected based on relevance to CVD, kidney disease, as well as metabolic disorders. Furthermore, we discuss how these factors put a disproportionate burden on the Indonesian health system and should be looked up as a unified clinical as well as public health challenges, rather than separate entities. Addressing CKM syndrome in Indonesia requires early detection in primary care, integration and collaboration programs, as well as multi-sectoral approaches to reduce the progression of CKM syndrome. The lessons from Indonesia may provide insight for other low middle-income countries (LMICs) undergoing similar conditions.

## 1 Introduction

Chronic kidney disease (CKD), cardiovascular disease (CVD), and metabolic risk factors cumulatively can result in multiorgan dysfunction and increase the risk for major adverse cardiovascular events and mortality. This condition is known as cardiovascular-kidney-metabolic (CKM) syndrome (1). The introduction of the term CKM syndrome aims to provide a multidisciplinary approach to prevention and therapy by developing strategies based on a range of stages, starting with stage 0 (no risk factors), progressing to stage 1 (dysfunctional/excess adiposity), stage 2 (metabolic risk factors or moderate-high risk CKD), stage 3 (subclinical CVD), and stage 4 (presence of CVD) (1, 2). In addition, the stages also reflect the hypothesis that dysfunctional/excess adipose tissue (stage 1) can be a predisposing factor for other metabolic diseases and CKD through mechanisms of inflammation, oxidative stress, insulin resistance, and vascular dysfunction (stage 2), which then leads to a very high risk of renal and cardiovascular events (stages 3–4) (3, 4).

The link between cardiovascular, kidney, and metabolic problems is a public health concern in the United States and became the leading cause of mortality in the United States in 2021 (2, 3, 5). The prevalence of CKM syndrome reached 79.5%, 31.3%, 41.6%, and 6.7% in stages 1, 2, 3, and 4, respectively, with overweight, obesity, hypertension, hypertriglyceridemia, and diabetes being the most prevalent risk factors (3, 6). In Asia, specifically South Korea, the prevalence of CKM syndrome is highest in stage 2 (43.4%), subsequent to stage 1 (25.4%), stage 0 (21.1%), stage 3 (7.3%), and stage 4 (2.8%), with risk factors frequently found in patients notably hypertension, diabetes, and dyslipidemia (7).

Although data on the prevalence of CKM syndrome in Indonesia are undetermined, the prevalence of risk factors for CKM syndrome, such as hypertension, diabetes, dyslipidemia, and obesity, is comparable to the respective countries of the United States and South Korea. According to recent data, Indonesia has encountered a rising burden of risk factors and CKM syndrome manifestations. Cardiovascular disease is a leading cause of death in Indonesia, accounting for 37–38% of all mortality, with strokes representing the most prevalent cause, followed by ischemic heart disease and diabetes mellitus (8, 9). According to the 2020 National Health Insurance report, the overall costs of CVD reach 10.3 trillion rupiah (USD 664.5 million), establishing it as the disease with the highest financial burden. Chronic kidney disease, another risk factor and manifestation for CKM syndrome, is also a public health concern in Indonesia. The estimated incidence is 973 instances per million people, which above the global norm (10). At the same time, the burden of metabolic risk factors, such as diabetes, which contribute to the development of CKD and CVD, is expected to rise from 10.9% in 2018 to 11.3% in 2023, and obesity, which was 13.6% in 2018 to 14.4% in 2023 (11–13). These findings collectively highlight the critical need for integrated prevention and treatment strategies that simultaneously address several cardiovascular, kidney, and metabolic risk factors in Indonesia’s healthcare system (14, 15).

In this study, the perspective synthesizes secondary data drawn from national and international reports, including the Indonesian Basic Health Research (RISKESDAS) 2013 and 2018, Indonesian Health Survey (SKI) 2023, BPJS Health (National Health Insurance) reports 2020, medical professional organizations in Indonesia such as Indonesian Renal Registry by the Indonesian Nephrology Association, The Indonesian Society of Endocrinology, as well as the Institute for Health Metrics and Evaluation (IHME), International Diabetes Federation (IDF), American Diabetes Association (ADA), World Heart Federation (WHF), American Heart Association (AHA), Kidney Disease: Improving Global Outcomes (KDIGO), World Health Organization (WHO), and some hospital registries in Indonesia. The selection of sources was based on the extent to which it represented metabolic, renal, and cardiovascular diseases in Indonesia. The data were then narratively evaluated and integrated to highlight actual epidemiological trends as well as policy implications for Indonesian settings.

## 2 Multidirectional relationship of metabolic disorders, chronic kidney disease, and cardiovascular diseases in cardiovascular-kidney-metabolic syndrome

CKM syndrome has been characterized by dysfunction or excessive adipose tissue. This condition has been associated with the release of detrimental pro-inflammatory and pro-oxidative products into the vascular, cardiac, and renal tissues, and could possibly reduce insulin sensitivity (16). CKM syndrome is classified into five stages: stage 0 is a condition without risk factors, stage 1 is a condition with excess adiposity/dysfunction but no signs of CKD or CVD, either subclinical or clinical, stage 2 is the presence of metabolic risk factors or CKD with moderate-high risk, stage 3 manifests as subclinical CVD, and stage 4 is a condition with clinical CVD or CKD stage 4 based on KDIGO staging (1, 16).

In addition to metabolic risk factors, several other factors can accelerate the progression of CKM syndrome, including chronic inflammatory conditions, a family history of diabetes or kidney disease, mental health and sleep disorders, elevated levels of high-sensitivity C-reactive protein, and in women, premature menopause, poor pregnancy outcomes, polycystic ovary syndrome, and men who suffer erectile dysfunction (4, 16).

Racial, ethnic, and genetic factors also influence the development of CKM syndrome. For example, even with the same BMI, South Asian men and women have greater ectopic fat reserves in the liver than White Europeans (17). Furthermore, a study of over 40,000 participants from South Asian and White European populations found that South Asians have higher visceral, liver, and muscle fat accumulation but lower muscle volume than Europeans (18). In Indonesia, increasing waist circumference was linked with worsening dysglycemia status in both men [median, IQR; 75.0, 69.2–81.5 vs. 75.6, 70.0–83.2 vs. 78.2, 71.0–86.8 for normal, prediabetic, and diabetic populations, respectively, *p*-value for trend <0.001] and women [median, IQR; 78.0, 70.0–85.0 vs. 79.0, 71.0–87.0 vs. 82.0, 72.3–90.0 for normal, prediabetic, and diabetic populations, respectively, p-value for trend <0.001] (19). These differences also serve as the foundation for the development of a more data-driven consensus suited to Asian populations, such as the obesity consensus in South and Southeast Asia, which has a different definition and cutoff point than the worldwide generalization (20). As a result, the scope and definition of CKM syndrome in Indonesia are compiled based on its demographic profile, characteristics, as well as the laboratory references (Supplementary Table 1).

## 3 Overview of CKM syndrome in Indonesia

### 3.1 Prevalence of metabolic disorders in Indonesia

Metabolic disorders are one of the early components in the pathophysiology of CKM syndrome. The metabolic risk factors of metabolic syndrome, namely central obesity, dysglycemia, dyslipidemia, and hypertension, have various pathological consequences for the body such as endothelial dysfunction, atherogenesis, thrombosis, myocardial infarct, fibrosis, and cardiac remodeling (4, 21). Metabolic syndrome has been linked with an increased risk of developing various subtypes of CVD, including coronary heart disease (CHD), peripheral artery disease (PAD), cardiac arrhythmia, and heart failure (HF). The progression from metabolic syndrome to type 2 diabetes is thought to involve pancreatic beta-cell dysfunction in chronic insulin resistance, which can lead to increased risk of vascular and renal disease (4).

**Figure 1:**
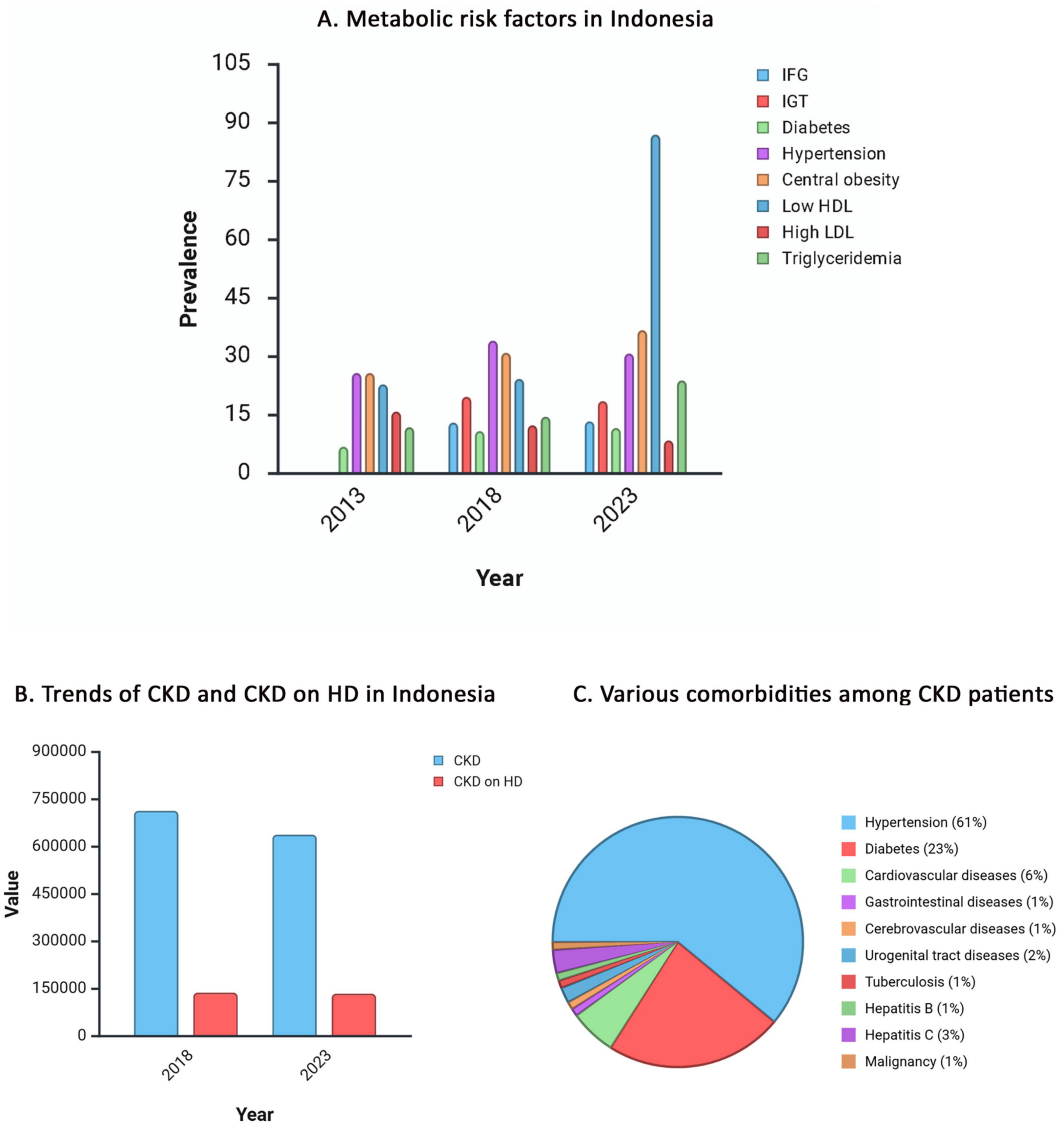
The trends of emerging risk factors for CKM Syndrome in Indonesia. **(A)** Prevalence of metabolic risk factors in Indonesia in 2013, 2018, and 2023. The increasing trend is observed in diabetes (6.9% in 2013, 10.9%in 2018, 11.7% in 2023), central obesity (26.6% in 2013, 31% in 2018, 36.8% in 2023), and triglyceridemia with high and very high level (11.9% in 2013, 14.6% in 2018, 23.9% in 2023). The slightly decreasing trend is observed in high and very LDL levels (15.9% in 2013, 12.4% in 2018, 8.5% in 2023), while low HDL is shown an increasing trend (22.9% in 2013, 24.3% in 2018, 87% in 2023). Hypertension shown to be increased from 2013 to 2018 (25.8 to 34.11%), but slightly reduced in 2023 (30.8%). Furthermore, IFG and IGT prevalence in 2013 were not available, but the trend from 2018 to 2023 shown to be very little difference from 13.1 to 13.4% for IFG and 19.7 to 18.6% for IGT. **(B)** The trends of CKD patients and CKD patients on HD in Indonesia. **(C)** The common comorbidities among CKD patients with hemodialysis increased in 5-year trend with the most common comorbidity among patients are hypertension, diabetes, and CVD. Created with biorender.com. CKD, chronic kidney disease; CKM, cardiovascular-kidney-metabolic; CVD, cardiovascular disease; HD, hemodialysis; HDL, high density lipoprotein; IFG, impaired fasting glucose; IGT, impaired glucose tolerance; LDL, low density lipoprotein.

Overall, the prevalence of metabolic syndrome in Indonesia is reported to reach 21.66% with the most common main components being low high density lipoprotein (HDL) levels (66.41%), hypertension (64.45%), central obesity (43.21%), and insulin resistance (0.82%) (21). In line with this, data from RISKESDAS also shows that the incidence of various metabolic disorders in Indonesia is quite worrying, especially the incidence of diabetes mellitus, central obesity, and triglyceridemia, which have experienced an increasing trend over the past decade. According to the 2013 and 2018 RISKESDAS, as well as the 2023 SKI, diabetes mellitus has increased every 5 years, from 6.9% in 2013 to 10.9% in 2018 and 11.7% in 2023. Similarly, central obesity has risen from 26.6% in 2013 to 31% in 2018 and 36.8% by 2023. Triglyceridemia with high and very high level of triglyceride has also reportedly increased, from 11.9% in 2013 to 14.6% in 2018 and 23.9% in 2023. Impaired fasting blood glucose (IFG) and impaired glucose tolerance (IGT) also showed comparable increases, from 13.1% in 2018 to 13.4 in 2023 for IFG, while IGT is expected to decrease from 19.7% in 2018 to 18.6% in 2023. Hypertension has had a fluctuating trend, rising from 25.8% in 2013 to 34.11% in 2018, and then decreasing to 30.8% by 2023. Meanwhile, high low density lipoprotein (LDL) and low HDL cholesterol levels have been observed to fluctuate results. High and very high LDL cholesterol levels were reported as: were reported as: 15.9% in 2013, 12.4% in 2018, and 8.5% in 2023. Low HDL cholesterol increased from 22.9% in 2013 to 24.3% in 2018, with a sharply rise of 87% in 2023 (Figure 1A) (11–13). According to the International Diabetes Federation, the rate of diabetes mellitus in Indonesia in the 20–79 years age range is estimated to continue to increase from 11.3% (20,426 people) in 2024 to 12.6% (28,584 people) in 2050 (22).

Significant disparities have been observed across various demographic groups in Indonesia. A study comprising more than 8,500 individuals from 20 provinces and diverse ethnic backgrounds found that women experience metabolic syndrome nearly twice as commonly as males (29.83% [95% CI: 28.52–31.14] vs. 11.81% [95% CI: 10.80–12.83]) (21). A cross-national comparison between Indonesian and the Netherlands showed a similar pattern with women in Indonesia having a greater rate of metabolic syndrome than men (46% vs. 28%), whereas in the Netherlands the rate is 24% for women and 36% for males. Among Indonesians, women are more likely to have central obesity (57.5% vs. 16.8%), poor HDL cholesterol (45.3% vs. 28.6%), and hypertension (64.6% vs. 56.2%) (23). Moreover, urban residents in Indonesia have a higher prevalence of metabolic syndrome than rural residents (25.19% [95% CI: 23.92–26.45] vs. 17.72% [95% CI: 16.54–18.89]), while the age factor did not show a significant difference between middle age (40–65 years), 21.05% (95% CI: 20.38–22.30), and age (>65 years), 23.29% (95% CI: 21.00–25.15) (21). These findings reveal significant geographical and sex disparities in metabolic syndrome in Indonesia.

Compared to other low middle-income country (LMIC) countries such as Myanmar, the prevalence of diabetes seems to decrease. In 2011, the prevalence of diabetes mellitus in Myanmar was around 7.1%, but by 2024 it had dropped to 6.7%, with prediabetes, including IFG, at 7.2% and IGT at 10.1% (24). Another study in Nigeria found that the prevalence of diabetes mellitus was only 2% in 2017 and will rise to 3% by 2024 (25, 26). In Indonesia, however, the prevalence of diabetes has risen in recent decades. Different trends can be attributed to a variety of factors, including socioeconomic circumstances, lifestyle changes, and the implementation of policies in each country. For instance, the government of Myanmar plans to decrease the prevalence of diabetes by 20% in 2030. Myanmar has taken several efforts, including providing training, implementing continuing medical education programs, developing guidelines and standard operating procedures, and educating people about medical condition through multimedia. Furthermore, inter-ministerial coordination is underway to minimize sugar and salt-rich meals, alcohol, cigarettes, and their derivatives, and saturated fat consumption (27).

A general inability to identify early symptoms is one of the problems that needs to be addressed in order to lower the number of metabolic disorder cases. According to the 2023 Indonesian Health Survey, only 2.2 percent of people aged 15 and older had diabetes mellitus diagnosed by a physician, compared to 11.7% who had a diagnosis through blood sugar measurement (13). The disparity between these numbers highlights the need for early detection and raising public awareness of diabetes mellitus. The high prevalence of prediabetes, which is shown by impaired glucose tolerance (IGT) (18.6%) and impaired fasting plasma glucose (IFG) (13.4%), may lead to challenges as well. According to ADA, prediabetes is a major risk factor for diabetes, CVDs, and a variety of other cardiometabolic consequences. Prediabetes is also linked to obesity (particularly central or visceral obesity), dyslipidemia (high triglycerides and/or low HDL cholesterol), and hypertension (28). Another study also supported this statement that at least 25% of people with prediabetes develop diabetes within 5 years, and 70% get diabetes over their lifetime (29). A 6-year follow-up study of more than 1,000 Indonesian patients reported that the incidence of cardiovascular events in pre-diabetic and diabetic population reached 9.7% (30).

Diabetes mellitus has been strongly associated with kidney damage, particularly diabetic nephropathy. A significant percentage, ranging from 20 to 40%, of individuals diagnosed with diabetes is predicted to develop diabetic nephropathy (31). This occurs due to chronic hyperglycemia which causes progressive damage to the renal microvasculature (32). Individuals with diabetes mellitus also have a 2–4 times higher risk of cardiovascular disease compared to individuals without diabetes (33). Cardiovascular problems in diabetes are caused by hyperglycemic circumstances that directly affect endothelial function and promote and accelerate the development of atherosclerosis (34). Furthermore, hyperinsulinemia can activate a number of inflammatory signaling pathways, promoting the onset and progression of atherosclerosis (35).

### 3.2 Prevalence of chronic kidney disease in Indonesia

As one of the endpoints of CKM syndrome (i.e., Stage 4b), end-stage renal disease (ESRD) is a serious medical condition. This is because ESRD can increase morbidity and mortality, particularly through cardiovascular complications (36, 37). Patients with CKD are at increased risk for cardiovascular problems such as CHD, HF, atrial fibrillation (AF), and sudden death. The incidence among CKD and CVD is directly proportional to CKD stage; the higher the stage, the greater the risk of CVD. CKD is a chronic and systemic proinflammatory condition that leads to vascular and myocardial remodeling in atherosclerotic lesions, vascular calcification and vascular senescence, as well as myocardial fibrosis and heart valve calcification. CKD has been linked with features of accelerated cardiovascular system aging and increased cardiovascular mortality (4, 38).

The prevalence of chronic renal disease in Indonesia has declined over the last 5 years, from 0.5% in 2018 to 0.2% in 2023, but the number of patients requiring hemodialysis continues to rise (Figure 1B) (12, 13). Other data from the Indonesian Renal Registry reflect this finding, with the number of hemodialysis patients increasing from 22,140 in 2020 to 157,929 in 2022, representing 63,489 new patients (39).

Data from the 2023 Indonesian Health Survey revealed notable demographic disparities among individuals with CKD. Men exhibited a higher prevalence than women (0.22% [95% CI: 0.19–0.24] vs. 0.14% [95% CI: 0.12–0.16]). The prevalence also increased with age, peaking in the 55–64 age group (0.40%), 65–74 age group (0.45%), and those aged ≥75 years (0.57%). Residential differences were less pronounced, although patients in urban areas showed a slightly higher prevalence than those in rural areas (0.19% [95% CI: 0.17–0.22] vs. 0.15% [95% CI: 0.13–0.18]) (13). The most common comorbidities among Indonesian CKD patients are hypertension (61%), diabetes mellitus (23%), and CVD (6%) (Figure 1C) (39). This is consistent with KDIGO guidelines, which state that people with hypertension, diabetes, or CVDs are at high risk for CKD (40). These findings suggest the interconnectedness between demographic and comorbid factors in shaping CKM syndrome progression (stages 3–4) and emphasize the importance of targeted, integrated management strategies.

In comparison to Thailand, CKD patients were 17.5% (about 11.6 million), with 8.6% (5.7 million) in advanced stages (stages 3–5) and more than 0.1 million requiring dialysis (41). In Pakistan, the total prevalence of CKD among all age groups was 21.2%, with diabetes mellitus (27.1%), unknown etiology (16.6%), and hypertensive nephropathy (15.2%) being the most common risk factors (76). Meanwhile, Indonesia has a lower prevalence of CKD than other LMIC countries. This is most likely due to an inadequate level of early detection and reporting, with many cases being misdiagnosed (underdiagnosed) because CKD is commonly asymptomatic (42).

### 3.3 Prevalence of cardiovascular disease in Indonesia

According to data from the 2023 Indonesian Health Survey, the prevalence of CVD among men and women was nearly equal (0.80% [95% CI: 0.75–0.84] vs. 0.91% [95% CI: 0.86–0.95]). The risk of CVD also increased with age, with particularly substantial increases happening in the 65–74 age range (4.05%) and those older than 75 (4.60%). Environmental conditions in urban markedly increase the incidence of CVD compared to rural areas (1.08 [95% CI: 1.03–1.13] vs. 0.53 [95% CI: 0.49–0.57]) (13). Furthermore, a study by Maharani et al., which assessed the risk of cardiovascular disease in more than 20,000 individuals in the Malang area, East Java, Indonesia, found that the risk of high CVD was higher in urban areas than semi-urban and rural areas (31.6% [95% CI: 30.7–32.5] vs. 28.7% [95% CI: 27.3–30.1] vs. 26.2% [95% CI: 25.2–27.2], respectively) (43).

Globally, CVD mortality rates are reported to be declining, but in Indonesia, the condition continues to increase (44). In the last 30 years, the number of deaths from CVD in Indonesia has increased twofold, from 292,000 in 1990 to 659,000 in 2019 (45). According to a World Heart Federation report, the percentage of CVD deaths in Indonesia has reached 41.1%, with smoking (31%), air pollution (19.3%), and obesity (16.6% in women, 6.5% in men) as the most common risk factors (46). Compared to Laos, CVD deaths in Laos reached 33%, with smoking accounting for 27.9%, air pollution for 20.4%, and alcohol consumption for 12% in men and 10.6% in women (47). The high burden of CVD in LMICs can be explained by the epidemiological transition theory, which is a change in disease patterns due to demographic factors (aging and population growth) as well as socioeconomic and behavioral changes such as the spread of a Western diet and decreased physical activity (48).

In clinical practice, the relationship between CKM syndrome risk factors and clinical outcomes (in particular, cardiovascular events) can be observed, however, these associations may not always exhibit causative impact. For example, a study conducted at Dr. Cipto Mangunkusumo National Referral Hospital in Jakarta found that in-hospital major adverse cardiac event (MACE) occurred among patients with hypertension (47.3%), diabetes (40%), and chronic kidney disease (66.7%) (49). In line with this, a study conducted at Dr. Soetomo General Hospital in Surabaya, East Java, Indonesia, found that patients diagnosed with acute coronary syndrome (ACS) had a history of hypertension (51%), dyslipidemia (77%), and diabetes (60%) (50). Furthermore, a retrospective cohort study at Dr. Wahidin Sudirohusodo General Hospital, a referral hospital in Sulawesi, reported that major adverse cardiac and cerebrovascular events (MACCE), especially HF, in patients with non-ST-elevation myocardial infarction (NSTEMI) had a significantly higher incidence in patients with a history of DM than patients who had just been diagnosed with DM (40.31% vs. 12.40%, OR 2.26, 95% CI: 1.06–4.82, *p* = 0.034) (51).

Hypertension is strongly associated with CVD risk in Indonesia (43, 52, 53). Lifestyle modifications, such as gaining ideal body weight, altering a bad diet (rich in sodium and low in potassium), increasing physical exercise, and quitting smoking and alcohol intake, can all help to reduce hypertension (54). The 2023 Indonesian Health Survey (SKI) reported a hypertension incidence of 34.1% in 2018 and 30.8% in 2023. Despite this decline, only 8.6% reported being diagnosed by a medical professional, while one-third (30.8%) were diagnosed based on blood pressure measurements. Furthermore, more than half of hypertensive patients in Indonesia reported not taking their medication on a regular basis (36.4%) or not taking any medication at all (16.9%). As a silent disease, the majority of hypertensive patients in Indonesia (62.8%) reported feeling healthy and thus requires no treatment (13).

Although hypertension is the most common risk factor, it is rarely a stand-alone illness. Other risk factors that coexist with hypertension, such as dyslipidemia, diabetes, obesity, and others are co-present with hypertension and have been linked with the development of CVD (52, 55). For example, in Indonesia, high LDL cholesterol in men and diabetes mellitus in women are strongly connected to higher CVD mortality, particularly ischemic heart disease (46, 52).

Obesity and being overweight are strongly correlated with higher cardiovascular death rates (52, 56). As previously stated, obesity can increase other major risk factors for CVD, such as elevated blood glucose, the development of metabolic syndrome and diabetes, elevated blood pressure or hypertension, worsening lipid profiles, particularly triglycerides, and lowering cardioprotective HDL cholesterol, as well as increased systemic inflammation, all of which increase the risk of major CVD (57). In Indonesia, the prevalence of overweight, obesity, and central obesity is 14.4, 23.4, and 36.8%, respectively. Obesity is also three times more common in diabetics and hypertensives aged 18 to 59 years in Indonesia (13). This demonstrates the overlapping situation of numerous risk factors for CKM syndrome, which are likely interrelated and may contribute to cardiovascular problems (stages 3–4 of CKM syndrome).

## 4 Management strategies for the CKM syndrome in Indonesia

It is possible to forecast that the condition of CKM syndrome in Indonesia will exhibit a rising trend over time when considering the high prevalence of metabolic disorders, CKD, and CVDs as previously explained. In Indonesia, there are no established recommendations for managing CKM syndrome; however, a number of preventive and curative measures have been put in place to address cases of non-communicable diseases (NCDs) in general.

**Figure 2:**
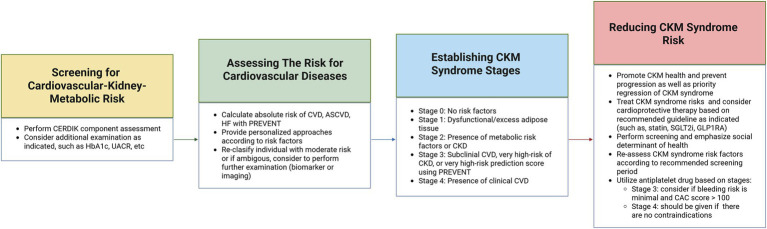
Framework for the management of CKM syndrome in Indonesia. The first step is to screen the metabolic risk of CKM syndrome, followed by assessing the CVD risk, continue with establishing the stage of CKM syndrome, and finally reducing the risk of CKM syndrome. Created with biorender.com. ASCVD, atherosclerotic cardiovascular disease; CAC, coronary artery calcium; CKD, chronic kidney disease; CKM, cardivascular-kidney-metabolic; CVD, cardiovascular disease; GLP-1RA, glucagon-like peptide-1 receptor agonist; HbA1c, hemoglobin A1C; HF, heart failure; PREVENT, predicting risk of cardiovascular disease events; SGLT2i, sodium glucose co-transporter 2 inhibitors; UACR, urine albumin-creatinine ratio.

As a professional organization in Indonesia consisting of internal medicine specialists with various specializations, the Indonesian Society of Internal Medicine (PAPDI) comprehensively reviewed the management of CKM syndrome in Indonesia. Through collaboration between subspecialty professions, integration of NCDs management by the Indonesian government, and elaboration of current guidelines on CKM syndrome, metabolic disorders, CKD, and CVD (4, 28, 40, 58–62), the framework for managing CKM syndrome in Indonesia was developed as shown in Figure 2.

In terms of clinical implications, a multidisciplinary care pathway has been developed to manage the clinical outcomes of CKM syndrome, particularly in acute and critical cases such as CODE STEMI and CODE STROKE. Several major hospitals in Indonesia have implemented this protocol, including Dr. Cipto Mangunkusumo Hospital in Jakarta, which serves as the national referral hospital, Universitas Indonesia Hospital in Depok, West Java, and Hospital in Aceh, Indonesia (63–65). A more coordinated and integrated diagnosis and management approach is used in this protocol. Research shows that this program is beneficial because it considerably lowers mortality, MACE, and door-to-balloon time in ST-elevation myocardial infarction (STEMI) patients (66).

Several government initiatives in Indonesia, initiated by the Ministry of Health (MoH) in collaborate with National Health Insurance (JKN), have supported in the prevention of chronic diseases including the CKM syndrome. For example, the Integrated Community Health Post (Posbindu), a health facility-level program under the direction of government-funded community health centers (known as Puskesmas) that provides early detection services, focusing on, including hypertension and diabetes mellitus, the Healthy Living Community Movement (Gerakan Masyarakat Hidup Sehat), a national community movement to promote healthy, as well as CERDIK campaign which stands for frequent health checkups, cigarette smoke cessation, regular physical activity/exercise, a healthy and balanced diet with less sugar, salt, and fat, adequate rest, and stress management (67, 68). Furthermore, the government developed the policy of the Healthy Indonesia Program with a Family Approach (Program Indonesia Sehat dengan Penilaian Keluarga), which provides comprehensive health services using a family risk approach in primary care, and the Chronic Disease Management Program (Program Penanggulangan Penyakit Kronis, known as PROLANIS), a chronic disease management program in primary care that focuses on patients with hypertension and diabetes (68).

One of the programs that most closely aligns with the principles of CKM syndrome screening is PROLANIS, which focuses on managing chronic conditions including diabetes mellitus and hypertension (69). Monthly medical consultations, peer-group education from medical professionals, reminders for healthcare center visits, peer-club activities involving walking and exercise, home visits, routine health status monitoring (including blood glucose testing for patients with diabetes mellitus), and laboratory evaluations for metabolic control and kidney function every 6 months are just a few of the advantages that this program offers (70). A number of examinations, such as cardiac biomarkers or imaging, body mass index (BMI), waist circumference, lipid profile, fasting glucose or HbA1c, serum creatinine, UACR, and estimated glomerular filtration rate (eGFR), should be part of PROLANIS screening in order to assess CKM risk (1, 4). The PROLANIS program has been shown to have a good impact, although it has limited coverage, and several studies consider its effectiveness as low (68, 71). Some of the causes of this problem include disparities in healthcare worker in primary health care facilities who lack a clear understanding of chronic disease management, a lack of budget and optimal human resources to run the program, the program’s exclusivity, which applies only to JKN members, and a range of geographic conditions and participant specific needs that is unable to met, resulting in unequal service quality (71).

Recently, the government has demonstrated its efforts through new policy breakthroughs such as the One Health (Satu Sehat) digital platform, which integrates health information from all members of Indonesia’s digital health ecosystem, including healthcare facilities, regulators, payers, and digital service providers (72). Furthermore, every Indonesian is eligible for a free health examination program on their birthday and for 30 days afterward (73). Although still in progress, this program is viewed as showing the government’s dedication and efforts to promote holistic patient care, including for patients with CKM syndrome.

Given the challenges that the Indonesian healthcare system encountered in carrying out CKM syndrome management, several government policies must be adjusted and directed to comprehensively manage CKM syndrome. The policy implications include addressing patients’ socioeconomic determinants of health (SDOH), which broadly defined as the conditions in which people are born, grow, live, work, and age, as well as their access to power, money, and resources (74). Economic stability, access to and quality of education, access to healthcare facilities, residential environment, and social and community context are all SDOH components that should be investigated (58, 74, 75). Identifying each of these components allows the government to develop measures that lower the risk of CKM syndrome. Furthermore, the government can strengthen primary healthcare providers’ ability to prevent and manage CKM syndrome by increasing human resources, establishing specialized CKM syndrome clinics or teams that can be integrated with the PROLANIS clinic, and providing CKM syndrome training, all of which must be included in each region’s measurable and targeted strategic plans. The government can additionally bridge the integrated referral strategy for CKM syndrome from primary to advanced facilities by providing a specific diagnostic package under JKN, allowing payments to be paid in the same way that other existing diagnoses are. Moreover, the addition of supporting examinations within the screening system, which can be integrated with PROLANIS, should be prioritizes the principle of inclusivity, meaning it should not only target a specific age group or be included in the JKN program. Integrated surveillance and monitoring on the One Health (Satu Sehat) digital platform can be accessed by multidisciplinary healthcare workers for CKM syndrome, including primary healthcare physicians, cardiologists, nephrologists, endocrinologists, nutritionists, and others.

At the level of practical strategies, primary health care workers can raise awareness of the GERMAS and CERDIK campaigns, collaborating with families, communities, non-governmental organizations (NGO), and even the government itself (67, 68). Furthermore, by increasing the primary health care system’s human resources and capacity (i.e., Puskesmas), specialized teams or clinics focusing on NCDs and CKM syndrome can be established. The team of CKM Syndrome in Puskesmas can start the screening process by conducting low-cost and simple examinations, such as body mass index, waist circumference, random blood glucose, and others. Physician at both the primary and secondary care levels can utilize the predicting risk of cardiovascular disease events (PREVENT) calculator for risk stratification for CKM syndrome. The PREVENT calculator considers blood glucose and liver function tests, smoking habits, medication use, and gender, as well as age risk factors. The calculator is suitable for those aged 30 to 79 and can predict the likelihood of a heart attack, stroke, and kidney failure up to 10 and 30 years in advance (75). More importantly, the establishment of an integrated referral system for CKM syndrome cases will make it easier for primary care physicians to refer patients if they identify conditions requiring further examination and management, including when calculating PREVENT scores with intermediate to high results.

Another practical recommendation at the hospital level is to develop a CKM syndrome unit with a multidisciplinary team that includes nephrologists, cardiologists, and endocrinologists, as well as nutritionists, public health specialists, and other relevant professionals. Furthermore, models such as CODE STEMI and CODE STROKE can be used to create a therapeutic pathway system, ensuring collaboration between divisions while managing overlapping comorbidities.

This perspective aims to provide information and awareness regarding CKM syndrome in Indonesia. The active participation of all parties in managing the CKM syndrome in Indonesia is critical. The involvement of the government, community, and appropriate professional organizations may collectively have a substantial impact on the management of CKM syndrome. As the information on the prevalence of CKM syndrome in Indonesia is still very limited, this analysis relies on secondary data from various sources which have different sampling frame and diagnostic criteria. For instance, data from Riskesdas and SKI were mostly used to illustrate the representation of risk factors for CKM syndrome. Data gathering techniques included examinations, measurements, and interviews. In contrast, various clinical outcomes of CKM syndrome in hospitals were demonstrated using data from retrospective studies with variable sample sizes and significant clinical outcomes such as STEMI, stroke, and MACE. While this combination of sources provides a comprehensive picture of the disease’s progression from risk to outcome, differences in population coverage, diagnostic methods, and case severity should be considered when drawing conclusions. Therefore, further studies on CKM syndrome in Indonesia, particularly longitudinal CKM syndrome registry and cost-effectiveness studies related to CKM syndrome, are urgently needed to expand knowledge and information about this condition.

## 5 Conclusion

CKM syndrome represents a complex interrelationship between metabolic disorders, CKD, and CVD. The burden of CKM syndrome in Indonesia is reflected in the high incidence of metabolic disorders, CKD, CVDs, and other risk factors. Management of CKM syndrome requires a holistic, comprehensive, and collaborative approach, ranging from prevention, screening, and individualized management to the formulation of public policies that prioritize SDOH in CKM syndrome.

## Data Availability

Publicly available datasets were analyzed in this study. This data can be found here on the available links below: The National Institute of Health Research and Development, Ministry of Health, Republic of Indonesia, including: Basic health research report 2013, available online at: https://repository.badankebijakan.kemkes.go.id/id/eprint/4428/; main findings of basic health research 2018, available online at: https://repository.badankebijakan.kemkes.go.id/id/eprint/3514/; Indonesian health survey 2023, available online at: https://www.badankebijakan.kemkes.go.id/hasil-ski-2023/; and The Indonesian Renal Registry Report 2022, available online at: www.indonesianrenalregistry.org.
