# Supplementary material for: Cardiovascular-kidney-metabolic syndrome in Indonesia: a commentary on the need for integrated management and policy action
Source: Front Public Health. 2026 Jan 13;13:1707551. doi: 10.3389/fpubh.2025.1707551 (PMC12838263; doi:10.3389/fpubh.2025.1707551)
Supplement: Supplementary file 1 [file Table_1.docx]

Supplementary Table 1. The scope and definition of CKM syndrome in Indonesia

| **Stage** | **Scope and definition** |
| --- | --- |
| Stage 0: No risk factors for CKM syndrome | Healthy individuals: normal BMI and waist circumference, normoglycemia, normotension, normal lipid profile, no subclinical/clinical CKD/CVD. |
| Stage 1: Excess/dysfunctional adipose tissue | Fulfill > 1 criteria:   - BMI > 23 kg/m^2^ - Waist circumference > 80/90 cm for women/men, respectively - FBG > 100-125 mg/dL or 2hPPG 140-199 mg/dL or OGTT 140-199 mg/dL or HbA1c 5,7-6,4% - LDL >130 mg/dL without prediabetes, and prediabetes >100 mg/dL |
| Stage 2: Metabolic risk factors and CKD | Metabolic risk factors include:   - LDL > 30 mg/dL - Triglyceride > 150 mg/dL - Hypertension with BP >120/80 mmHg - Metabolic syndrome - Diabetes   Or presence of CKD |
| Stage 3: Subclinical CVD | Subclinical ASCVD or HF in individuals with stage 1/2. Diagnosis is established by:   - Subclinical ASCVD by measurement of CAC - Subclinical HF through elevated cardiac markers, MRI/echocardiography, or a combination of both.   Stage 3 equivalent risk:   - Very high-risk CKD (Stage G4 or G5 or very high-risk according to KDIGO) - High 10-year predicted CV risk |
| Stage 4: Presence of CVD | Clinical CVD (coronary heart disease, heart failure, stroke, peripheral artery disease, and atrial fibrillation) |
| 2hPP = 2-hour post prandial blood glucose; ASCVD = atherosclerotic cardiovascular disease; BMI = body mass index; BP = blood pressure; CAC = coronary artery calcium; CKD = chronic kidney disease; CKM = cardiovascular-kidney-metabolic; CV = cardiovascular; CVD = cardiovascular disease; FBG = fasting blood glucose; HbA1c = hemoglobin A1c; HF = heart failure; KDIGO = kidney disease improving global outcomes; LDL = low density lipoprotein; OGTT = oral glucose tolerance test; MRI = magnetic resonance imaging | |
